# Intestinal Bacteria Encapsulated by Biomaterials Enhance Immunotherapy

**DOI:** 10.3389/fimmu.2020.620170

**Published:** 2021-02-10

**Authors:** Yilun Liu, Zhongmin Li, Yuanyu Wu, Xiabin Jing, Lin Li, Xuedong Fang

**Affiliations:** ^1^ Department of Gastrointestinal Colorectal and Anal Surgery, China-Japan Union Hospital of Jilin University, Changchun, China; ^2^ Changchun Institute of Applied Chemistry, Chinese Academy of Sciences, Changchun, China

**Keywords:** intestinal bacteria, probiotic, immunotherapy, immune cell, biomaterial, oral delivery

## Abstract

The human intestine contains thousands of bacterial species essential for optimal health. Aside from their pathogenic effects, these bacteria have been associated with the efficacy of various treatments of diseases. Due to their impact on many human diseases, intestinal bacteria are receiving increasing research attention, and recent studies on intestinal bacteria and their effects on treatments has yielded valuable results. Particularly, intestinal bacteria can affect responses to numerous forms of immunotherapy, especially cancer therapy. With the development of precision medicine, understanding the factors that influence intestinal bacteria and how they can be regulated to enhance immunotherapy effects will improve the application prospects of intestinal bacteria therapy. Further, biomaterials employed for the convenient and efficient delivery of intestinal bacteria to the body have also become a research hotspot. In this review, we discuss the recent findings on the regulatory role of intestinal bacteria in immunotherapy, focusing on immune cells they regulate. We also summarize biomaterials used for their delivery.

## Introduction

In the human intestine, there are more than 100 trillion bacterial cells that mainly exhibit commensalism with the host (1, 2) and play a role in the maintenance of host health (3). In this commensalistic relationship, intestinal bacteria not only participate in the regulation of the host immune system and the promotion of bone marrow hematopoiesis but also regulate the maturation and function of hematopoietic cells originating from the yolk sac (4, 5). Moreover, intestinal bacteria can regulate the barrier function *via* interaction with epithelial cells and stromal cells (6–8). The functions of intestinal bacteria widely range from local to systemic levels, including metabolism regulation, hematopoiesis, inflammation, immunity, and other physiological functions (8–11). However, changes in intestinal ecology can disrupt this commensalistic relationship. For instance, some symbiotic bacteria called pathobionts might cause, or even worsen, a number of diseases (10, 12, 13). Increasing evidence has demonstrated that dysbiosis has been connected to various diseases, including tumors, viral infection, inflammatory bowel disease (IBD), diabetes, and liver cirrhosis (10, 14, 15).

Owing to their impact on various human diseases, intestinal bacteria have been recently receiving increasing attention. Aside from their pathogenic effects, intestinal bacteria also exert beneficial effects in reducing gastrointestinal inflammation, preventing colorectal cancer, and treating some diseases (16–19). The mechanisms by which intestinal bacteria affect inflammation, immunity, and local therapeutic response have also been elucidated (8, 20, 21). Currently, intestinal bacteria, including *Escherichia coli* (22, 23), *Bifidobacterium* (24, 25), *Filamentous fungus* (10), *Lactobacillus* (26), *Bacillus subtilis* (27), and *Bacteroides fragilis* (28, 29), have been applied in the therapy of diseases such as diabetes (30), gastrointestinal diseases (31, 32), and allergic diseases (33, 34). Interestingly, intestinal bacteria can also potentiate antitumor therapies (35–37). With the advancements in precision medicine, intestinal bacteria have thus become increasingly important in the treatment of various diseases. Thus, studies aiming to understand the factors influencing intestinal bacteria and the strategies for their manipulation to enhance therapeutic efficacy are increasing (36).

Traditional tumor therapies, such as chemotherapy, surgery, radiation therapy, and molecular targeted therapy, are the primary methods used to treat tumors at different stages (31). However, they have some drawbacks, such as toxic side effects and recurrence after treatment. With the advancement in tumor research, immunotherapy has emerged as a promising therapeutic modality (38). Compared with chemotherapy, immunotherapy of tumors has fewer side effects. In cases wherein immunotherapy is effective in patients suffering from tumors, it could prolong their survival period and even cure tumors clinically. Although immunotherapy has shown promising potential in the treatment of hematological and solid tumors, its efficacy remains limited due to the variability in immune responses and susceptibilities to tumor types among patients (39). Therefore, only some patients benefit from immunotherapy. Recent studies have shown that the regulation of intestinal bacteria can influence the effects of immunotherapy (35, 40, 41). Moreover, intestinal bacteria have been applied to the immunotherapy of many diseases, such as type 1 diabetes and IBD, and yielded remarkable outcomes (42, 43). This treatment approach that uses intestinal bacteria is called bacterial therapy.

To delineate the factors that affect intestinal bacteria and improve their immunotherapeutic effects, many studies have attempted to regulate intestinal bacteria using antibiotic treatment or fecal microbiota transplantation (FMT) (14, 44, 45). However, both methods can cause large-scale and holistic changes in abundance and diversity of intestinal bacteria. Therefore, to regulate the population of a certain bacterium or transfer some type of probiotics into the host for bacterial therapy, an efficient way of delivering intestinal bacteria is necessary. Compared with intravenous (i.v.) injection, the oral administration of bacteria can improve patient compliance and avoid the risk of systemic infections that might be caused by the i.v. injection. However, because the oral administration route involves the passage of bacteria through the stomach and gut, various bacterial activities may be compromised due to the presence of gastric acid and bile salts in the gastrointestinal system. Therefore, the design and selection of biomaterials for the encapsulation and delivery of intestinal bacteria are essential. Many biomaterials, including Eudragit (46), chitosan (47), and alginate (48), have been widely used to encapsulate and protect intestinal bacteria against acid, bile, and other harsh components. Moreover, the properties of these biomaterials, including permeability, mechanical stability, and pH sensitivity, have been specially designed to improve the survival of intestinal bacteria in the acidic environment of the stomach and ensure their complete release in the intestine (49, 50).

**Figure 1:**
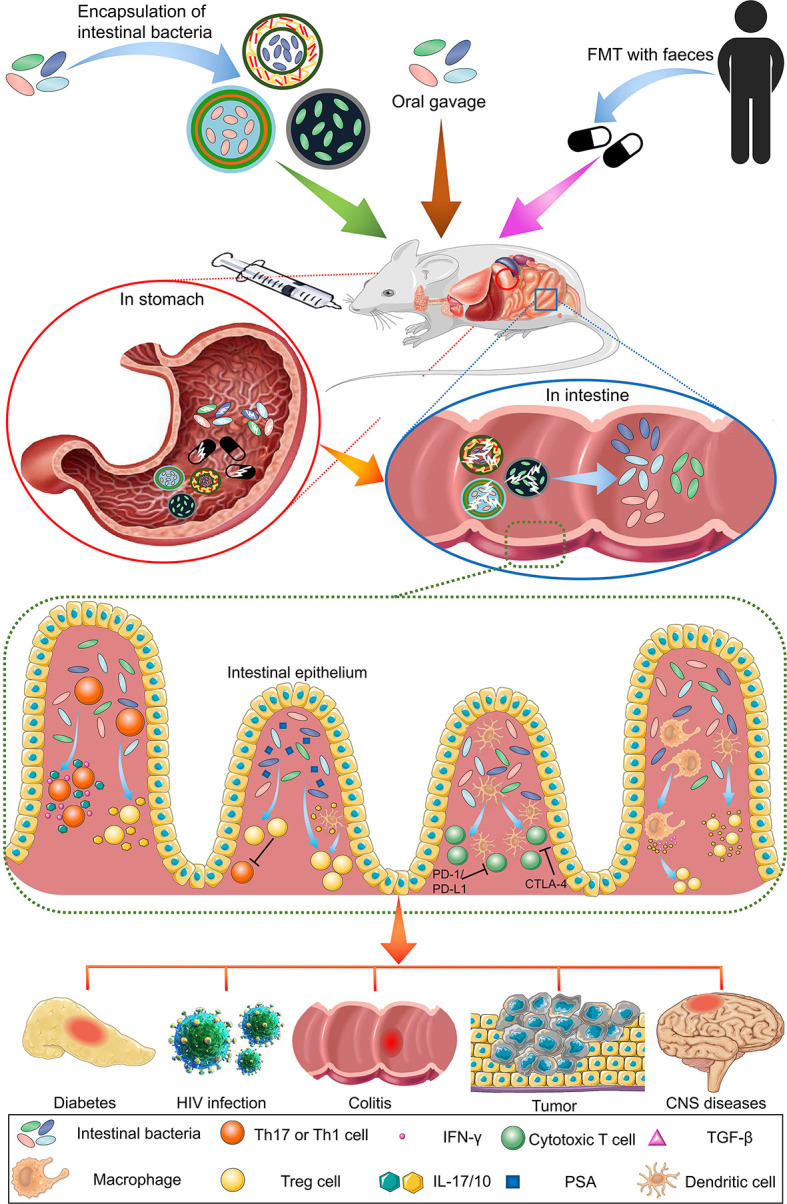
Overview of the oral administration of intestinal bacteria for immunotherapy in various diseases. Common intestinal bacterial delivery methods include oral delivery (gavage) and intravenous injection. Gavage is more widely used because of its safety profile. Compared with free bacteria and FMT, bacteria encapsulated by biomaterials can resist the acidic environment of the stomach, and their contents can be released in the intestine. The released bacteria exert immune regulation functions beneficial to the treatment of various diseases.

In this review, we focused on the immunotherapeutic effects of intestinal bacteria mainly exerted by regulating various immune cells, as well as the biomaterials used to encapsulate and deliver the bacteria possessing these regulatory functions. We first discuss various immune cells, which are classified according to their types, regulated by intestinal bacteria. Then, we summarize the recent progress on biomaterial encapsulation methods in various intestinal bacterial species. **Figure 1** schematically shows the oral administration of intestinal bacteria and their immunoregulatory function in various diseases.

## Regulating Immunotherapy by Affecting Immune Cells

The immune system of the human body has a crucial impact on the development of various diseases. Diseases, such as inflammation and tumors, can alter the microenvironment of the body. For example, tumor cells in the tumor microenvironment (TME) have been reported to closely interact with the extracellular matrix (ECM) and stromal cells (51). A variety of immune and nonimmune cells that secrete cytokines and chemokines and express various surface receptors have been found in the TME. These cells have been shown to drive chronic inflammation and immunosuppression and promote the development of proangiogenic tumor environment (52). Immune cells, such as lymphocytes, dendritic cells (DCs), macrophages, and natural killer (NK) cells, are important in tumor development or suppression (53). A spatiotemporal dynamic analysis of 28 different kinds of immune cells that infiltrated tumors found that the composition of infiltrating immune cells changes at each tumor stage, with particular cells having a major impact on survival (54). Therefore, regulating these immune cells may improve the immunotherapy of tumors and other diseases.

Owing to some limitations in the application of immunotherapy (39, 55) and the regulation of the intestinal bacterial function (56, 57), many studies have combined the use of intestinal bacteria with immunotherapy, revealing their key role in regulating the response of immunotherapy, especially in tumors, by affecting immune cells (58–61). The findings of recent studies on bacteria used for immunotherapy and the immune cells they influenced are summarized in **Table 1**.

**Table 1:** Intestinal bacteria used for the immunotherapy of diseases.

Bacterial species	Disease	Immune cell target	Reference
*Lactobacillus* *Bifidobacteria*	Type 1 diabetes	Th17 cells	(56)
*segmented filamentous bacteria* (SFB)	Diabetes	Th17 cells	(62)
*Lactobacillus johnsonii strain* N6.2 (LjN6.2)	Type 1 diabetes	Th17 cells	(63)
*Lactobacillus casei*	HIV infection	Th1 cells	(64)
*Lactobacillus rhamnosus* GG (LGG) *Escherichia coli Nissle* 1917 (EcN) *heat-inactivated* VSL#3	Liver cancer	Th17 cells	(16)
*Bacteroides*	Inflammation	Tregs	(65)
*Bacteroides fragilis*	IBD	Foxp3^+^ Tregs	(43)
*L.paracasei* DSM 13434, *L.plantarum* DSM15312 and DSM 15313	Inflammation	Foxp3^+^ Tregs	(66)
*L.acidophilus, L.casei*, *Lactobacillus reuteri, Bifidobacteria* *Streptococcus thermophilus*	Inflammation	Foxp3^+^ Tregs	(67)
*Faecalibacterium* spp.	Melanoma	Cytotoxic T cells	(68)
*Bifidobacteria longum* *Collinsella aerofaciens* *Enterococcus faecium*	Metastatic melanoma	Cytotoxic T cells	(24)
*Enterococcus hirae* 13144 *(E. hirae)*	Tumor	Memory T cells	(45)
*Bacteroides fragilis*	Tumor	Memory T cells	(44)
*Bifidobacterium*	Tumor	Dendritic cells	(15)
*Bifidobacteria* LMG 13195	Inflammation	Dendritic cells	(69)
*Bacteroides fragilis*	IBD	Dendritic cells	(70)
*Bacillus subtilis* 7025	Tumor	Macrophages	(27)
SCFA	CNS diseases	Microglia	(71)
DNA of *Escherichia coli*	Tumor	B cells	(72, 73)
DNA of *Mycobacteria*	Tumor	NK cells	(74)

### Regulation of T-Cells by Intestinal Bacteria

T-lymphocytes, also called T-cells, are derived from the bone marrow. Following their differentiation and development in the thymus, they are distributed to immune organs and tissues through the blood circulatory and lymphatic systems, thus exerting their immune function. T-cells are critical in many diseases, including tumors (75). According to their function and surface markers, T-cells can be classified as helper T-cells, regulatory T-cells, cytotoxic T-cells, suppressor T-cells, and memory T-cells. In this section, we summarized the recent findings on the regulation of these T-cells by intestinal bacteria.

#### Helper T-Cells

Helper T-cells, whose major surface marker is CD4, play a role in intermediate immune response. They can proliferate and spread to activate other types of immune cells involved in direct immune response. Helper T-cells can be activated through an antigenic reaction with a polypeptide presented by the major histocompatibility complex II (MHC II). Once activated, they can secrete cytokines, regulate, or assist in the immune response.

Intestinal bacteria regulate immunotherapy for type 1 diabetes (T1D) *via* helper T-cells. A study analyzing the fecal samples of biobreeding diabetes-prone (BB-DP) and biobreeding diabetes-resistant (BB-DR) mice (56) found that the fecal matter of BB-DR mice is enriched in both *Lactobacillus* and *Bifidobacterium* species, whereas that of BB-DP mice is abundant in *Bacteroides*. The prevalence of *Bacteroides* in mice with T1D suggests that intestinal bacteria are involved in the occurrence and development of diseases. Furthermore, intestinal bacteria can regulate T-helper 17 (Th17) cells. Among non-obese diabetic (NOD) mice, female mice without segmented filamentous bacteria (SFB) showed a higher prevalence of diabetes, whereas those with SFB were resistant to diabetes (62). However, in male mice, there was no significant difference in the onset of diabetes between the two groups. To explore the relationship between SFB and diabetes, flow cytometry was performed on tissue derived from the small intestinal lamina and associated lymph nodes in SFB^+^ and SFB^-^ female mice. The results showed an evident induction of Th17 cells in the small intestinal lamina propria of SFB^+^ females. Another study has demonstrated that the oral administration of the *Lactobacillus johnsonii* strain N6.2 (LjN6.2) from BB-DR rats conferred T1D resistance to BB-DP rats, but that of *Lactobacillus reuteri* strains did not (76). This resistance of LjN6.2-fed BB-DP mice was due to a change in Th17 cells within the mesenteric lymph nodes (63), which was not observed in non-gut–draining axillary lymph nodes, indicating that the change in Th17 cells was caused by LjN6.2 interactions within the mesenteric lymph node. Overall, these studies indicate that the induction of T1D could be circumvented by the intestinal bacterial-mediated differentiation of Th17 cells.

**Figure 2:**
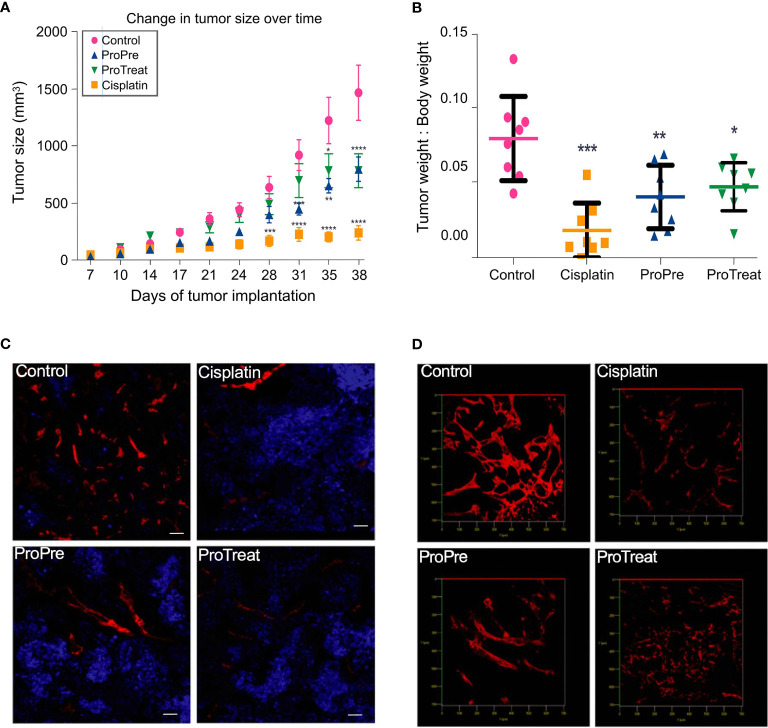
The Prohep intestinal bacterial mixture improves hypoxia in tumor tissues and reduces tumor volume. **(A)** Changes in tumor size during 38 d. **(B)** Tumor weight of each group after the experiment. **(C)** Immunostaining of representative tumor sections using the CD31 angiogenesis (red) and GLUT-1 hypoxia (blue) markers. **(D)** Images of a 3D model acquired after superimposing multiple confocal planes by confocal Z-stack imaging (section thickness of 25 μm). *0.01 < *P* value < 0.05; **0.001 < *P* value < 0.01; ***P value < 0.001. Adapted from ref. (16).

Most importantly, by affecting helper T cells, the intestinal bacteria can regulate immunotherapy for tumors. To investigate the potential mechanism underlying the inhibition of tumor progression and control of hepatocellular carcinoma (HCC) by intestinal bacteria feeding, Li and coworkers used Prohep to design their experiments (16). Prohep is a new mixture of intestinal bacteria comprising heat-inactivated VSL#3, viable *Escherichia coli* Nissle 1917 (EcN), and *Lactobacillus rhamnosus* GG (LGG) (1:1:1). Prohep not only significantly reduced tumor size and weight by 40% but also retarded tumor growth compared with the control (**Figures 2A, B**). In addition, after treatment with Prohep, the hypoxic region of the tumor was obviously increased, indicating that the reduction in tumor size might have been associated with cell death caused by hypoxia (**Figure 2C**). In **Figure 2D**, the confocal Z-stacks of 3D models show that compared with the control group, the number of vessel sprouts and the region of blood vessels in each tumor section were obviously decreased. The tumor group treated with intestinal bacteria had decreased number of Th17 cells. Moreover, metagenome sequencing revealed the association between intestinal bacteria and HCC development. Therefore, treatment with Prohep may promote the growth of beneficial bacterial colonies, including *Oscillibacter* and *Prevotella*, which are known producers of anti-inflammatory metabolites. Subsequently, these bacteria led to the reduction in the number of Th17 cells and promoted the differentiation of regulatory T-cells (Tregs; introduced in next section) in the intestine. In summary, tumor reduction induced by probiotic feeding relies on the downregulation of IL-17 and its major producer, Th17 cells.

Intestinal bacteria have also been found to prevent infections and suppress allergies. Chilba et al. demonstrated that a co-culture with Lactobacillus casei *in vitro* promoted the development of Th1 cells, resulting in an increased production of interferon γ (IFN-γ), and also stimulated CD11b+ cells to produce interleukin 12 (IL-12) in mouse spleen cells (77). Intestinal bacteria that increase the production of IL-12, such as *L. casei*, can effectively prevent infections. By controlling the balance of Th1/Th2 cells, *L. casei* has also been reported to suppress allergic diseases. This immunomodulation is partly due to the regulation of indoleamine 2,3-dioxygenase (IDO). IDO is widely distributed in the body and participates in tryptophan metabolism to produce 3-hydroxyanthranilic, quinolinic acid, and kynurenine (78). These three tryptophan degradation products have excellent blood–brain barrier penetration ability, which is a key obstacle to the application of many drugs to treat brain diseases (79). Moreover, 3-hydroxyanthranilic acid and quinolinic acid can selectively drive the apoptosis of Th1 cells, whereas kynurenines can promote the development of Tregs, suggesting that these metabolites may possess immunoregulatory effects (80). By expressing IDO and activating subsequent biological processes, the use of specific intestinal bacteria could also enhance the efficacy of immunotherapy for brain diseases.

#### Tregs

Tregs exhibit immunosuppressive functions, and their deficiency contributes to the development of allergies and autoimmune diseases; thus, they are T-cells that regulate autoimmune activity *in vivo*. In addition, Tregs expressing specific tumor-associated antigens have been found in patients with tumor and aggregate in several kinds of solid tumors, where they may play a role in protecting tumors from cytotoxic immune responses (81). In fact, there is a negative correlation between the survival rate of patients with tumors and Treg infiltration. Therefore, the regulation of Tregs could be a promising treatment strategy in many diseases.

Bacterial metabolites affect the occurrence and progression of inflammation *via* mediating the communication between intestinal bacteria and the immune system. For example, butyrate, a short-chain fatty acid (SCFA) secreted by intestinal bacteria, promotes the extrathymic production of Tregs (21), indicating that intestinal bacteria exhibit a regulatory function in the immunotherapy of inflammatory diseases, such as IBD. In a prospective study of patients suffering from metastatic melanoma treated with ipilimumab, Dubin et al. compared the composition of preinflammation fecal microbiota before and after the development of colitis (65). Increased *Bacteriodes* were found in the stools of participants resistant to colitis, whereas enriched *Faecalibacterium* and other *Firmicutes* were detected in those with high incidence of checkpoint-block-induced colitis (57). Moreover, enrichment in the genus *Bacteroides* was observed in patients with resistant colitis. As one of the primary bacterial species in the human gut, *Bacteroides* can suppress inflammation *via* stimulating the differentiation of Tregs (82). This immunomodulatory effect was consistent with the results obtained by Dubin et al. and Chaput et al. (57, 65).

In addition, immunotherapy can also be regulated by specialized bacterial molecules, such as the polysaccharide A (PSA) of *Bacteroides fragilis*. Round et al. demonstrated that the Toll-like receptor (TLR) pathway could be activated in T-cells by the human commensal bacterium *Bacteroides fragilis* to establish an interaction between the host and intestinal bacteria (28). Deficiency in TLR2, which is located on CD4^+^ T-cells, can promote antimicrobial immune responses, resulting in reduced colonization of the mucosa by *B. fragilis*. However, this can be recovered by the PSA of *B. fragilis* that directly activates TLR2 on Foxp3^+^ regulatory T-cells, leading to the production of mucosal tolerance. Meanwhile, *B. fragilis* not expressing PSA could not suppress the host immune response. Another study confirmed the regulatory function of the PSA of *B. fragilis* and showed that *B. fragilis* induced the development of Foxp3^+^ Tregs with unique inducible hereditary characteristics (43). Unlike naturally produced Tregs, these inducible Tregs were found in peripheral tissues, such as the intestine, rather than in thymic tissues and can secrete cytokines, such as interleukin 10 (IL-10) (83). Round et al. found that germ-free animals colonized with *B. fragilis* had increased inhibitory capacity of Tregs and produced only anti-inflammatory cytokines from Foxp3^+^ T-cells in the intestine (43). In addition, they showed that as an immunomodulatory molecule, the PSA of *B. fragilis* induced CD4^+^ T-cells into inducible Foxp3^+^ Tregs. These processes required TLR2 signaling to induce Tregs and express IL-10. More importantly, the PSA of *B. fragilis* was demonstrated to not only prevent but also cure experimental colitis in animals, supporting that intestinal bacterial molecules not only mediate the balance between health and disease but also regulate the efficacy of immunotherapy for many diseases.

After recognizing that changes in the composition of intestinal bacteria can influence inflammation, the potential mechanisms of intestinal dysfunction following HIV-1 infection became clearer. Early in 1990, researchers found that most patients with acquired immunodeficiency syndrome (AIDS) exhibited gastrointestinal disorders of variable severity (84); thus, they assumed that maintaining the integrity of intestinal ecology could improve the clinical outcomes of patients suffering from AIDS. Consequently, increasing evidence showed that probiotic treatment can protect the intestinal surface, delay the progression of HIV-1 infection to AIDS, and provide specific benefits in patients with HIV-1 infection (64, 85). The balance between Th17 cells and Tregs is an important immunoregulatory mechanism that influences the production of functional host immune responses to infection (86, 87). This balance is mediated by tryptophan catabolites, tryptamine, indole-3-aldehyde (IAld), indoleacetic acid, and indolelactic acid (ILA), which act as aryl hydrocarbon receptor (AHR) ligands and promote the differentiation of naive CD4^+^ helper T-cells into Tregs (88). Intestinal bacteria, such as *Lactobacilli* (89) and *Clostridium sporogenes*, can metabolize and convert tryptophan into the above-mentioned AHR ligands, thereby affecting the Th17/Tregs balance and exerting a beneficial effect on autoimmune diseases and inflammation. More specifically, intestinal bacteria inducing the differentiation of Th17 cells or upregulating Tregs can suppress the progression of inflammation and provide benefits in patients with viral infection. In addition, by expressing IDO and producing tryptophan metabolites, intestinal bacteria can activate the AHR signaling pathway and induce IL-22 production (90). IL-22 mainly fulfills intestinal barrier function and maintains the intestinal homeostasis by mediating mucosal host defense, thereby helping the body resist the invasion of intestinal pathogenic bacteria (91, 92). Overall, intestinal bacteria can indirectly up-regulate IL-22 to counter pathogenic bacterial infections and diarrhea caused by gut dysbiosis.

Kwon et al. identified a mixture of probiotics, designated as IRT5, comprising *L. casei*, *L. acidophilus*, *Bifidobacterium bifidium*, *L. reuteri*, and *Streptococcus thermophilus* (67). After its oral administration for 20 days, IRT5 upregulated CD4^+^ Foxp3^+^ Tregs and induced the hypo-responsiveness of T-cells. In addition, the transformation of T-cells into Foxp3+ Tregs was shown to be directly mediated by DCs (discussed in Section of DCs) specialized to express IDO as well as suppressor cytokines such as TGF-β and IL-10.

By regulating Tregs, intestinal bacteria can also affect the immunotherapy of allergic diseases, such as allergic asthma. Russelle et al. showed that changes in the intestinal bacteria due to antibiotics increase the susceptibility of neonatal mice to allergic asthma (93). Compared with streptomycin, vancomycin reduced intestinal bacterial diversity, especially that of *Bacteroides* and *Clostridiales*, and the reduced *Bacteroides* were replaced by abundant *Lactobacilli*. As previously discussed, species under the genus *Bacteroides*, such as *B. fragilis*, are associated with T-cell differentiation. Additionally, treatment with vancomycin decreased the number of Foxp3^+^ Tregs in the colon, but not in the lungs, following the destruction of *Clostridiales* population. Atarashi et al. reached the same conclusion and identified the *Clostridium* species as potent inducers of Tregs (94). However, the role of *Lactobacilli* remains controversial as the said study showed its negative correlation with Tregs, whereas another study showed that it induces Tregs (95). Therefore, there might be other unknown factors participating in the regulation of immunotherapy by intestinal bacteria or an unknown interaction between other *Lactobacilli* species or strains. The specific mechanisms, however, remain unexplored. As there are thousands of intestinal bacteria in the body, maintaining commensalism with the host, all intestinal bacteria in each organ should be seen as a whole when we regulate intestinal bacteria in order to influence immunotherapy. Based on this view, FMT has been applied at the microbial level to alter immune responses in the body and has shown good results in the regulation of immunotherapy.

#### Cytotoxic T-Cells

Cytotoxic T-cells are specific T-cells that secrete various cytokines and participate in immune function. They exert a killing effect on certain viruses, tumor cells, and other antigenic substances. In particular, cytotoxic T-cells and NK cells play an important role in defense against viruses and tumors. Recently, cytotoxic T-cells regulated by intestinal bacteria has been associated with the efficacy of the immune checkpoint inhibitors (ICIs).

ICI therapy leads to the activating of T-lymphocyte-mediated immune responses by inhibiting the interaction between T-cell inhibitory receptors and their homologous ligands on stromal cells or tumors (96). Currently, ICIs are mainly monoclonal antibodies targeting the programmed cell death protein 1 (PD-1)/PD ligand 1 (PD-L1) axis and cytotoxic T-lymphocyte antigen 4 (CTLA-4), and ICIs have achieved great success in the immunotherapy of tumors. However, ICIs cannot suppress tumor progression in most patients; thus, they often result in immune-related adverse events (irAEs) (97). The use of antibiotics could reduce the efficacy of ICIs in tumor immunotherapy, with ICIs showing excellent efficacy in the presence of specific intestinal bacterial species (98). For example, the immunostimulatory and antitumor effects of the CTLA-4 blockade was demonstrated to depend on the presence of various *Bacteroides* gut species (99). In patients suffering from renal cell carcinoma (RCC), advanced-stage non–small cell lung cancer (NSCLC), or bladder tumor treated with PD-1/PD-L1 blockade, the use of broad-spectrum antibiotics shortly before or after treatment was associated with adverse clinical outcomes (45).

**Figure 3:**
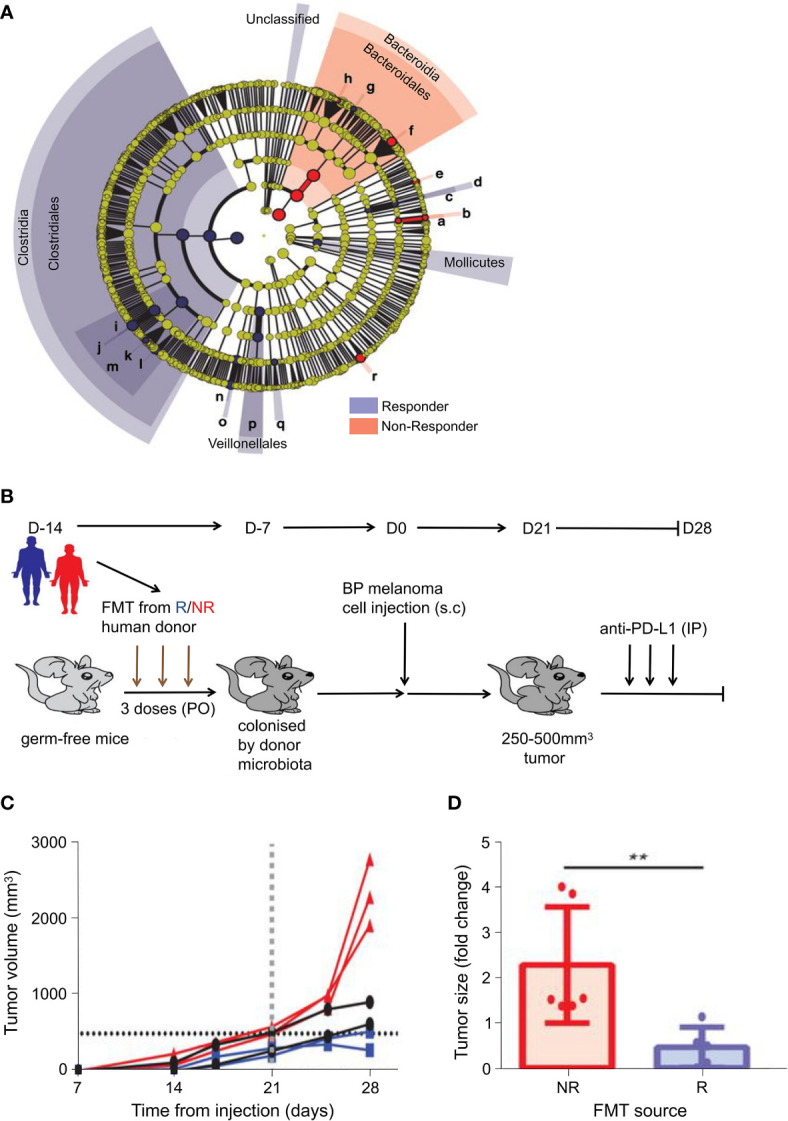
Differences in the composition of intestinal bacteria related to the effects of anti-PD-1 immunotherapy and antitumor immunity. **(A)** Taxonomic cladogram from LEfSe showing differences in stool taxa. The size of dot is positively correlated with the abundance of the taxon. Letters a–r represent the following taxa respectively: (a) *Gardnerella vaginalis*, (b) *Gardnerella*, (c) *Rothia*, (d) *Micrococcaceae*, (e) *Collinsella stercoris*, (f) *Bacteroides mediterraneensis*, (g) *Porphyromonas pasteri*, (h) *Prevotella histicola*, (i) *Faecalibacterium prausnitzii*, (j) *Faecalibacterium*, (k) *Clostridium hungatei*, (l) *Ruminococcus bromii*, (m) *Ruminococcaceae*, (n) *Phascolarctobacterium faecium*, (o) *Phascolarctobacterium*, (p) *Veilonellaceae*, (q) *Peptoniphilus*, (r) *Desulfovbrio alaskensis*. **(B)** Experiment designed to study the GF mice. Relative to days (indicated as D) of tumor injection (2.5 - 8 × 10^5^ tumor cells). **(C)** Tumor growth curves for each GF mouse from anti-PD-L1-treated R-FMT (blue, n = 2; median tumor volume = 403.7 mm^3^), NR-FMT (red, n = 3; median tumor volume = 2301 mm^3^), and Control (black, n = 2; median tumor volume = 771.35 mm^3^) mice. Statistics are as follows: p = 0.20 (R-FMT vs NR-FMT), p = 0.33 (NR-FMT vs Control) by the MW test. The black dotted line indicates the size limit of the tumor when treated with anti-PD-L1 (500 mm^3^). **(D)** Using the MW test, on the 14th day of implantation in NR-FMT mice (red) and R-FMT (blue), difference in tumor size expressed as fold change (FC) relative to the average tumor volume of Control GF mice. Data from 2 independent FMT experiments (R-FMT, n = 5, median FC = 0.18; NR-FMT, n = 6, median FC = 1.52). **P value < 0.01. Adapted from ref. (68).

Increasing evidences have demonstrated that intestinal bacteria can influence the effect of immune checkpoint blockade (ICB) treatment by regulating cytotoxic T-cells. Gopalakrishnan et al. observed notable differences in the composition and diversity of intestinal bacteria between responsive and non-responsive (NR) patients with melanoma receiving anti-PD-1 immunotherapy as shown in **Figure 3A** (68). Responsive patients had increased abundance of the *Ruminococcaceae* family (including *Faecalibacterium* spp.) compared to NR, and enrichment in *Faecalibacterium* spp. positively correlated with progression-free survival (PFS) and cytotoxic T-cell infiltration in the TME. To study the mechanism, FMT results confirmed the transferability of this phenotype. The detailed experimental design is presented in **Figure 3B**. Slower tumor growth and better immunotherapy efficacy were observed in mice subjected to FMT with stool samples from the responsive patients than in those from NR (**Figures 3C, D**). In a separate study, Matson and coworkers verified these findings using pretreated fecal samples from 42 patients with metastatic melanoma (24). *Enterococcus faecium*, *Collinsella aerofaciens*, and *Bifidobacterium longum* were found to be more enriched in responders. In addition, transferring fecal samples from the responders to germ-free mice has been shown to improve tumor control, enhance T-cell responses, and increase anti-PD-1 therapeutic effect. These effects are due to the regulation of cytotoxic T-cells by these intestinal bacteria.

#### Memory T-Cells

Memory and effector T-cells are formed following the division and differentiation of T-cells, respectively. Memory T-cells are of vital importance in recurrent immune responses. When the host immune system is invaded by the same antigen, memory T-cells remobilize the mechanisms used to kill the antigen previously. The identity of memory T-cells can be determined *via* the expression of CD45RA, CD27, and CD62L (100). Currently, memory T-cells are used in vaccines for infectious diseases (101) and have recently been found to exert an anti-tumor function.

**Figure 4:**
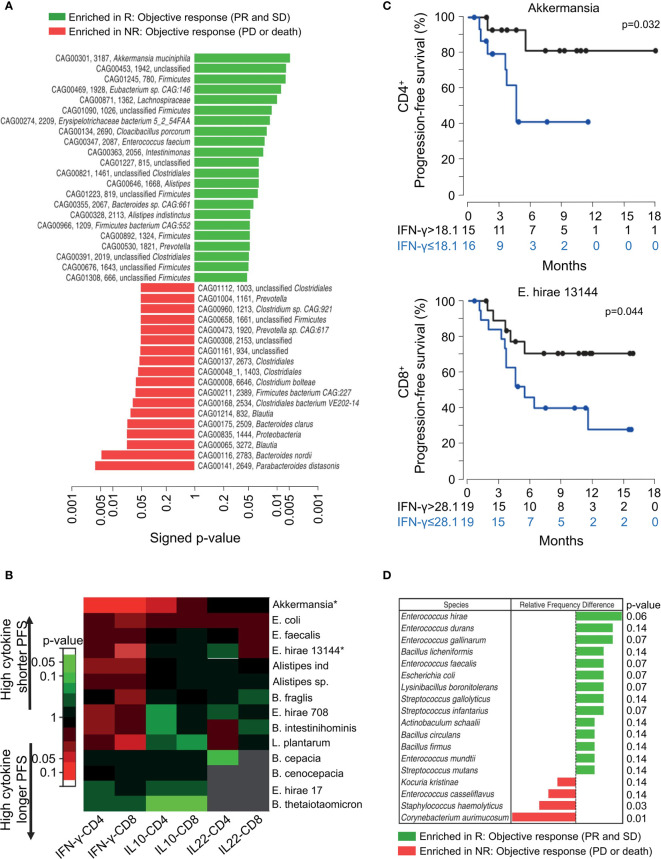
The composition of intestinal bacterial from stool samples determines the efficacy of PD-1 mAb therapy in cancer patients after 3 months. **(A)** Shotgun sequencing of stool samples at the time of diagnosis, using responders (R) (partial response or stable disease) determined according to the best clinical response relative to the non-responder (NR) (progress or death) of each MGS according to RECIST1.1 standard. P value of the entire cohort of n = 100 (60 patients with NSCLC and 40 with RCC). **(B, C)** Immune responses of circulating memory T-cells detected during PD-1 blockade and evaluation of the time to progression. **(B)** Heat map of the P values for each intestinal bacterium and each cytokine, classifying the PFS of patients with NSCLC RCC based on the median value of the production of cytokines in the entire cohort. Significant P values (<0.05, Student’s *t* test) are marked by asterisks as relevant intestinal bacteria. **(C)** Kaplan-Meier curves and Univariate analysis showing immune responses of PBS against peripheral blood memory Th1 and Tc1 directed against *A. muciniphila* and *E. hirae* 13144, respectively. **(D)** Stool samples of 16 R and 16 NR patients with NSCLC (defined as the best clinical outcome) analyzed based on culturomic before treatment; each intestinal bacterium having been identified by mass spectrometry. Colored bars show relative frequencies of each commensal in all stool cultures in R over NR patients, and the right graph shows P values with difference. *P value < 0.05. Adapted from ref. (45).

Routy et al. found a relationship among *Akkermansia muciniphila*, *Enterococcus hirae* 13144 (*E. hirae*), and memory T-cells (45). As shown in **Figure 4A**, patients with NSCLC and RCC responding to the PD-1/PD-L1 blockade had increased abundance of *A. muciniphila* in their feces than non-responders. They next investigated the responses of memory T-cells in the peripheral blood to the microbiota after initiating PD-1 blockade. **Figure 4B** presents the response of circulating memory CD4^+^ and CD8^+^ T-cells collected from 27 patients with NSCLC and 28 with RCC subjected to anti-PD-1 treatment. Due to the function of *A. muciniphila* and *E. hirae*, the responses of memory Th1 and Tc1 cells were enhanced in the responders, resulting in increased production of IFN-γ (**Figure 4C**). **Figure 4D** shows that based on 32 fecal samples, *E. hirae* was more abundant in responders with NSCLC than in non-responders. This further supports the relevance of probiotics, such as *E. hirae* and *A. muciniphila*, in predicting efficacious treatment.

In addition, Vetizou and coworkers demonstrate the modulatory function of intestinal bacteria in responses to anti-CTLA-4 therapy by regulating of memory T-cells (44). In both mice and humans, T-cell specific response to *B. fragilis* or *B. thetaiotaomicron* was related to the effect of the CTLA-4 blockade. Tumors in germ-free (GF) or antibiotic-treated mice did not respond to CTLA-4 blockade. However, after the adoptive transfer of *B. fragilis*-specific T-cells or gavage with *B. fragilis*, the presence of *Bacteroides* species determined the antitumor effect of the CTLA-4 blockade. Subsequently, the dynamics of the response of memory T-cells in humans and mice during CTLA-4 blockade was analyzed and revealed that T-cell response to anti-CTLA-4 therapy was due to the production of Th1 cells induced by specific memory T-cells. Moreover, the adoptive transfer of this specific type of memory T-cells into GF or patients with antibiotic-treated tumors could partially restore the efficacy of the immune checkpoint blocker.

### Regulation of Antigen Presenting Cells by Intestinal Bacteria

Antigen presenting cells (APCs) can absorb, process, and present antigens; hence, they constitute a key part of innate immunity. The major histocompatibility complex (MHC) class II molecule expressed on the surface of APCs can absorb pathogen proteins and help process them into short peptide segments, which are presented to T-cells. Thus, APCs are also known as the initiators of acquired immunity. The thymus-dependent antigen (TD-Ag) stimulated B-lymphocytes to produce antibodies, with the participation of not only T- and B-lymphocytes but also accessory cells. In general, APCs include macrophages, DCs, B–lymphocytes, and other cells that can express MHC class II molecules, the so-called full-time APCs. Other cells, such as the fibroblasts, endothelial cells, and various epithelial and mesothelial cells, also exhibit certain antigen presenting functions and are thus called non-full-time APCs. The expression of PD-L1 on DCs or macrophages might affect the efficacy of immunologic checkpoint inhibitors and therefore, in theory, could have the potential to predict the efficacy of drugs (102). We then speculate that intestinal bacteria could influence the efficacy of immunotherapy by regulating APCs.

#### DCs

Discovered by the Canadian scholar Steinman in 1973, DCs are the most powerful antigen-presenting cells at present. They were named for their dendritic or pseudopodal protrusions at their mature state. DCs can absorb, handle, and present antigens efficiently. Immature DCs show a strong ability to migrate, whereas mature DCs can effectively activate primary T-cells, which are essential for initiating, regulating, and maintaining immune responses. DCs have been closely linked with the occurrence and development of tumors. For instance, in the majority of solid tumors, higher DC infiltration correlates with better tumor prognosis. Effective antitumor immune responses mainly relies on the production of cellular immune responses based on CD8^+^ T-cells, which is also the basis of DCs in immunotherapy.

A previous study showed that C57BL/6 mice raised in Taconic Farms (TAC) or Jackson Laboratory (JAX) animal facilities showed differential colonization by segmented filamentous bacteria (SFB) (103). The difference in growth environment leads to the different composition of intestinal bacteria. Compared with JAX mice, an obvious enrichment of Th17 cells in TAC mice was observed. After 10 days of colonization of JAX-derived GF mice with SFB, the SFB-colonized lamina propria of both the small and large intestines were populated with increased Th17 cells. This suggests that intestinal bacteria, such as SFB, can stimulate immune response *via* direct contact with body tissues. However, further research found that SFB induced the production of serum amyloid A (SAA) by the terminal ileum, with SAA promoting the differentiation of Th17 cells under the action of DCs *in vitro*. The colonization of SFB led to the secretion of SAA, which further stimulated DCs in the intestine to induce the differentiation of Th17 cells, thus demonstrating that intestinal bacteria regulate host immunity through their secretory function.

In addition, substances with immunomodulatory functions, such as IDO, are not only regulated by intestinal bacteria (104, 105) but also expressed in immune cells, which makes the relationship between intestinal bacteria and immune cells more intricate. In fact, there are a series of human APCs that express IDO co-expressing cell surface markers CD123 and CCR6, by which they can be identified from other immune cells (106). In the family of DCs, IDO-expressing DCs can suppress inhibitory effector T cells and promote the differentiation of Tregs, proving to be beneficial in patients with autoimmune diseases like IgA nephropathy (IgAN) (107).

**Figure 5:**
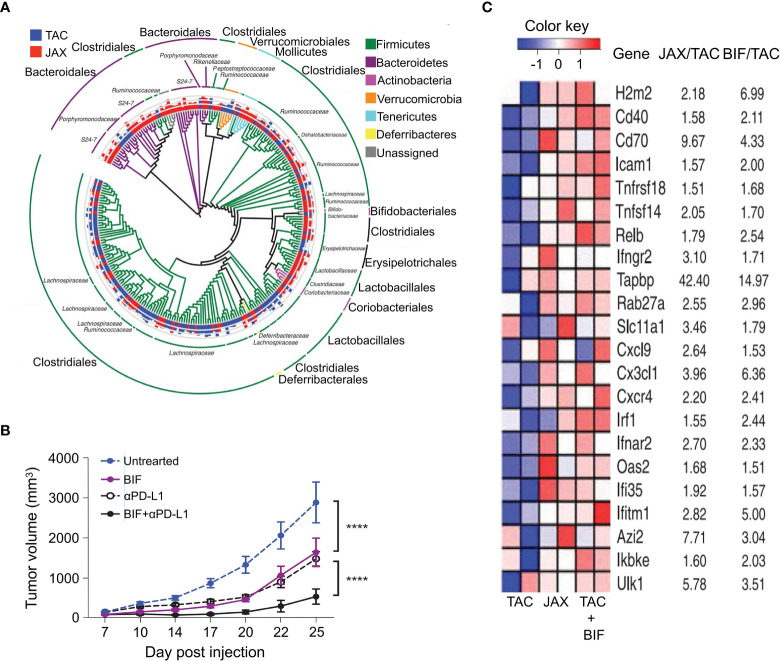
Tumor patients can benefit from direct administration of *Bifidobacterium*, which was shown to improve the DC cell-related tumor-specific immunity and effect of anti-PD-L1 monoclonal antibody treatment. **(A)** In newly obtained TAC and JAX mice, the phylogenetic analysis of taxa with obviously different abundance FDR < 0.05 (nonparametric t test); bars represent log-transformed fold changes, inner circle, log10(10); middle circle, log10(100); outer circle, log10(1000). **(B)** 7 and 14 d after the implantation of B16.SIY tumor, the tumor growth kinetics in TAC mice, untreated or treated with *Bifidobacterium*, anti-PD-L1 mAb 7, 10, 13, and 16 d after tumor implantation, or both regimens. **(C)** Heat map of key antitumor immunity genes in DCs isolated from untreated TAC, *Bifidobacterium*-treated TAC, and JAX mice. Mean fold change for each gene transcript is shown on the right. ****P value < 0.0001. Adapted from ref. (15).

Sivan et al. studied the growth of melanoma in TAC and JAX mice with different commensal intestinal bacteria and observed differences in spontaneous antitumor immunity (15). Particularly, they found that as the most distinct intestinal bacterial species between the two groups, *Bifidobacterium* (**Figure 5A**) unexpectedly enhanced antitumor immunity *in vivo*. *Bifidobacterium* alone or combined with anti-PD-L1 treatment effectively inhibited tumor growth (**Figure 5B**). Additionally, after its oral administration, the antitumor activity of CD8^+^ T-cells was improved, and it was attributed to DC-induced accumulation of enhanced CD8^+^ T-cells in the TME. **Figure 5C** displays that the key genes related to antitumor immunity in DCs that are significantly enhanced by the administration of *Bifidobacterium*. Notably, only live *Bifidobacterium* produced this effect, suggesting that *Bifidobacterium* colonized intestinal niches that enabled them to interact with host cells that regulate DCs or to systemically release soluble factors that enhanced the function of DCs. In addition, *Bifidobacterium* was eliminated in CD8^+^ T-cell-depleted mice, indicating that the regulatory function of *Bifidobacterium* relied on the activity of cytotoxic T-cells. Therefore, the regulation between intestinal bacteria and immune cells is mutual. Furthermore, *Bifidobacterium* can activate other immune regulatory pathways. For instance, *Bifidobacterium* LMG 13195 or their membrane vesicles promoted the differentiation of immature T-cells into CD25^+^ Foxp3^+^ Treg cells by acting on DCs and inducing the production of IL-10 *in vitro* (69). *Bifidobacterium* has been extensively studied and can be detected in the intestines after the first meal. It is also a typical probiotic due to its health promoting functions. As a member of the Bifidobacterium family, *Bifidobacterium* LMG 13195 is safe for human consumption. Therefore, *Bifidobacterium* LMG 13195 has the potential as a safe and effective adjuvant for immunotherapy in clinical practice.

Intestinal bacteria were also found to affect the immunotherapy of IBD by regulating DCs. As one of the most potent anti-inflammatory cytokines, IL-10 is required for protection during inflammation. In an experimental colitis model, researchers found that the PSA of *B. fragilis* protected subjects from inflammatory disease *via* promoting the proliferation of IL-10-producing CD4^+^ T-cells (70). Unlike other polysaccharides, PSA can be internalized by APCs and subsequently presented to T-cells, along with MHC class II molecules. Furthermore, the PSA of *B. fragilis* executed this process by regulating bone-marrow-derived DCs (BMDCs). Therefore, PSA may play a protective role during inflammation. The immunomodulatory capacity of symbiotic factors, such as PSA, may thus provide new treatment approaches for human inflammatory diseases.

In summary, the immunoregulatory role of DCs is multifaceted: DCs can inhibit immunity and exert a beneficial effect on autoimmune diseases such as IgAN, while they can also activate immune responses for immunotherapy of other diseases including tumors. Therefore, intestinal bacterial species that can regulate DCs may have diverse therapeutic applications. However, increasing attention must be paid to the changes in DCs mediated by pathogenic intestinal bacteria.

#### Macrophages

Macrophages have many functions and are important targets in the study of cell phagocytosis, cellular immunity, and molecular immunology. In vertebrate animals, macrophages are known to be involved in specific (cellular immunity) and nonspecific (innate immunity) defense. Their main function is to engulf pathogens and cellular debris. In addition, macrophages can activate multiple immune cells to release a variety of cytokines (108) and also regulate the differentiation and mobilization of neutrophils *via* secretion of granulocyte-colony stimulating factor (G-CSF) (109). Through these cytokines, macrophages can modulate the activity of other immune cells, such as neutrophils.

As we mentioned before, the PSA of *B. fragilis* provided protection against inflammatory disease *via* the production of the IL-10 cytokine. The relationship between macrophages and IL-10 was further demonstrated by Denning et al. In the lamina propria, some macrophages expressed several anti-inflammatory molecules, including IL-10; however, even after stimulation by Toll-like receptor ligands, the macrophages expressed only low levels of proinflammatory cytokines (110). The differentiation of Foxp3^+^ Tregs could be induced by these macrophages *via* a mechanism relying on the exogenous transforming growth factor-β, retinoic acid, and IL-10. Although this study did not use intestinal bacteria to regulate the immune process, the regulation of IL-10 by *B. fragilis* or the PSA they produce has been mentioned before. For example, Round and coworkers showed that the transformation of CD4^+^ T-cells into Foxp3^+^ Tregs that produce interleukin 10 (IL-10) is mediated by the PSA of *B. fragilis* (43). Therefore, we speculate that the PSA of *B. fragilis* synergizes with macrophage activity to enhance their immunomodulatory functions. In another study, the nucleoprotein fraction (NPF) of *Bacillus subtilis* 7025 cultural medium filtrate stimulated immune responses and exhibited antitumor effects (27). As NPF is mainly composed of histone and protamine, the NPF of *Bacillus subtilis* 7025 cultural medium filtrate has sufficient biological safety and can potentially be used in antitumor immunotherapy as it displays significant immunostimulatory effects through enhanced IFN-γ activity. Moreover, as one of the major cytokines involved in antitumor protection, IFN-γ can activate macrophages.

Remarkably, special macrophages called the microglia have also been reported to be regulated by intestinal bacteria. Microglia, which are found in the brain, are macrophages of the central nervous system (CNS) mainly involved in CNS diseases. Following the stimulation of inflammation, the antigenicity of microglia is enhanced. Host intestinal bacteria have been found to be vital in microglial homeostasis as microglia in GF mice exhibited obvious defects, changes in cell ratio, and immature phenotypes, resulting in impaired innate immune responses (71). In addition, less complex intestinal bacteria can lead to microglia defects, whereas the reconstitution of their complexity can partially restore the characteristics of microglia. Studies on SCFAs, which are bacterial fermentation products, found that SCFA influences microglial homeostasis and can be pathogenic to the CNS (111).

#### B-Cells

Progenitor cells of B lymphocytes (B-cells) are found in the island of hematopoietic cells in fetal liver. During development, B-cells are produced and differentiate in the bone marrow. B-cells can differentiate into plasma cells under antigenic stimulation, which can then synthesize and secrete antibodies, mainly involved in humoral immunity.

Intestinal bacterial DNA has been reported to have immunostimulatory effects on B-cells, thereby inducing the production of various cytokines critical in anti-tumor immunity (112). In 1991, researchers found that bacterial DNA could induce significant antibody responses in mice (72). When highly purified ssDNA collected from *E. coli* was used to stimulate lymphocytes, a dose-dependent response was observed, indicating lymphocyte proliferation. As the consumption of T-cells was not observed to reduce the proliferation of lymphocytes, bacterial DNA may have directly triggered the proliferation of B-cells. Another study showed that in bacterial DNA, unmethylated CpG dinucleotides rapidly activate B-cells to secrete IL-6 and IgM (73).

#### Other Immune Cells

The DNA of intestinal bacteria can activate NK cells and thus enhance antitumor immunity (74, 113). The administration of mycobacterial DNA can induce the secretion of interferon and enhance the activity of NK cells, resulting in tumor regression (114). An *in vitro* experiment showed that the nucleic acid-rich component from Bacille Calmette-Guerinvaccine (BCG) enhanced the activity of NK cells in mouse spleen, induced antiviral activity, and induced macrophages to produce cytotoxic factors against tumor cells.

Group 3 innate lymphoid cells (ILC3) are tissue-resident lymphocytes abundant in mammalian intestine and play an important regulatory role in intestinal inflammation and homeostasis (115). ILC3 proliferation has been associated with *L. paracasei* abundance *(* 116 *).* This phenomenon was also observed in the lungs after viral infection, subsequently suppressing the inflammatory response to achieve a therapeutic effect *via* mediating the proliferation of Th2 cells (117).

## Biomaterials for the Delivery of Intestinal Bacteria

In the previous section, we have introduced the effects of intestinal bacteria on the function of various types of immune cells, which ultimately enhanced immunotherapeutic effect in many diseases. For example, FMT is usually the preferred method to study of intestinal bacteria as a whole. When intestinal bacteria are regulated using FMT, their integrity can be maintained, and the influence of different bacteria on the regulation of the immune function does not need to be considered. In addition, the transfer of fecal samples from experimental mice to GF mice can not only reconstruct the composition of the intestinal bacteria but also facilitate the study of its effect on disease treatment.

However, FMT experiments cannot reveal the mechanisms and the role of certain intestinal bacteria in the regulation of immunotherapy. Therefore, to study the functions of several specific strains, single bacteria or combinations of several probiotics are delivered by oral administration (gavage) (26, 44) or intravenous injection (118). Intestinal bacteria can be delivered directly to the host through intravenous injection, gavage, and even anal perfusion (119, 120); however, these methods have certain disadvantages. Intravenous injection might lead to a variety of infectious diseases, such as bacteremia and septicemia. Especially in bacterial infectious diseases, there is a high risk of bacteria contaminating the blood. Thus, intravenous injection is rarely used in the studies we have discussed here. Anal infusion is more inconvenient than gavage and reduces patient compliance. Besides, intestinal bacteria are widely distributed in the colon. Therefore, the proper delivery of the target bacteria to various parts of the colon to simulate the situation of bacterial distribution in the human body is essential. However, anal perfusion can only deliver the target intestinal bacteria to areas near the descending colon and rectum and not the whole intestine. Thus, oral administration is the most widely used method for the delivery of intestinal bacteria.

Direct oral administration allows the passage of intestinal bacteria through the esophagus, stomach, small intestine, and colon in the proper order. However, this process involves the serious loss in bacterial activity due to the strong acidic environment in the stomach (pH = 2) and bile salts in the intestine (121, 122). This limitation might thus restrict the recognized roles of intestinal bacteria in promoting immunotherapy. Therefore, encapsulating intestinal bacteria with suitable materials conferring them protection and allowing their targeted release in the colon would be an effective method to address this issue.

Due to the strong acidic environment in the stomach, the materials used for bacterial encapsulation should be acid-resistant to maintain the integrity of the microcapsules during their travel to the colon. In addition, these materials should be biocompatible and automatically degraded in the colon to ensure host safety. For these purposes, various biomaterials, including alginate (123), enteric polymer (124), chitosan (125), and pectin (126), have been designed for the encapsulation and effective delivery of intestinal bacteria (122). **Table 2** summarizes these biomaterials.

**Table 2:** Biomaterials used for the oral delivery of intestinal bacteria.

Biomaterials	Bacteria	Encapsulating method	Reference
Calcium alginate/protamine (CAP)	*Lactobacillus casei*	Extrusion	(127)
Microcrystalline cellulose (MCC), calcium cross-linked alginate and lactose	*Lactobacillus casei*	Extrusion Eudragit coating	(128)
Sodium caseinate (SC) Fat sodium caseinate (FSC)	*Lactobacillus casei*	Emulsification	(129)
Alginate Fenugreek Gum Locust Bean Gum	*Pediococcus pentosaceus* KID7 *L. plantarum* KII2 *L. fermentum* KLAB6 *L. helveticus* KII13	Extrusion	(130)
Pea protein isolate–alginate capsules (PPCs)	*Lactobacillus reuteri* ATCC 53608	Extrusion cross-linking	(131)
Alginate–milk microspheres	*Lactobacillus bulgaricus*	Extrusion	(132)
Enteric polymer films Bile adsorbent resins	*Salmonella Typhimurium* SL3261	polymer film laminate (PFL)	(133)
Ethylcellulose Eudragit L100 55	*Bifidobacterium breve* NCIMB 8807	Polymer Film Laminate (PFL)	(124)
Cellulose Calcium carbonate Ca-alginate	*L. Plantarum*	Extrusion	(134)
Alginate-CNC-lecithin microbeads	*Lactobacillus rhamnosus* ATCC 9595	Freeze-drying	(135)
Alginate-silica microcapsules	*Lactobacillus rhamnosus* GG	Electrospraying Mineralization	(136)
Ethylenediaminetetraacetic- calcium-alginate	*Lactobacillus rhamnosus* ATCC 53103	Emulsification	(137)
Liposomes	*Escherichia coli*	Inverse-emulsion	(138)
Alginate-chitosan-alginate (ACA) microcapsules	*Escherichia coli* DH5	Electrostatic interactions	(139)
Cellulose microgels (CMs) Alginate	*Lactobacillus plantarum*	Extrusion	(140)
Pectin-starch hydrogels	*L.plantarum* ATCC 13643	Extrusion	(126)
Corn starch	*L. plantarum* 299v	Freeze-drying	(141)
Alginate Chitosan coating	*L. plantarum*	Extrusion	(142)
Alginate-chitosan	*Bifidobacterium longum*	Surface coating	(125)
Alginate-chitosan	*Bacillus coagulans* (BC)	Electrostatic interactions	(143)
Alginate/poly-llysine/pectin/poly-l-lysine/alginate (APPPA)	*Lactobacillus reuteri*	/	(144)
Pea protein-polysaccharide	*Bifidobacterium*	Extrusion	(145)
Alginate-chitosan	*Bifidobacterium breve*	Surface coating Layer-by-Layer	(146)
Alginate-chitosan	*L. plantarum* PBS067 *L. rhamnosus* PBS070 *Bifidobacterium animalis subsp. lactis* PBS075	Surface coating Emulsion	(147)

## Biomaterials for the Delivery of *Bifidobacterium*

*Bifidobacterium* is the most abundant bacterial genus in the intestines of breastfed infants (148) and is one of the main components of the gut microbiome associated with the maintenance of human health. The abundance and diversity of *Bifidobacterium* species, including *B. longum*, *B. breve*, and *B. adolescentis*, change throughout life. In the human intestine, *Bifidobacterium* levels decrease with age. In the body of infants and children less than 3 years old, the primary Bifidobacterial species is *B. breve*; in breastfed infants and young adults, the proportion of *B. adolescentis* gradually increases and becomes the main species; in the elderly, *B. longum* becomes the representative species (149, 150). In addition, these *Bifidobacterium* subpopulations exhibit specific health-promoting functions. For example, both *B. infantis* and *B. breve* in the intestine of infants prolonged the immune memory of vaccines (151). *B. longum* in adult intestines promoted immunotherapy response to tumors (24). Because of their excellent health promoting effects and wide applicability, many studies on the microencapsulation of *Bifidobacterium* have been conducted.

Yeung et al. designed a core–shell microgel consisting of an alginate core and a chitosan shell to encapsulate *B. longum* (125). Under aerobic storage and simulated gastric fluid (SGF), the viability and resistance of encapsulated *B. longum* were significantly improved. In 2015, Varankovich et al. developed protein–polysaccharide capsules for *B. adolescentis* (145). Compared with free bacteria, the established capsules provided significant protection to bacterial cells at 37°C in SGF. Moreover, when alginate or iota-carrageenan was used as polysaccharide, the capsules were easily dissolved, releasing 70–79% of bacterial cells into the simulated intestinal fluid (SIF) within 3 h. Moreover, when the capsules were freeze-dried, the number of live bacterial cells released was increased. Another study employed an alginate matrix for *B. breve* followed by alternate encapsulation using alginate and chitosan to coat bacteria using layer-by-layer (LbL) method (146). These multilayer-coated alginate matrices enhanced the viability of *Bifidobacterium breve* in a low-pH environment and delivered bacterial cells into the intestine, wherein their load was gradually released. Moreover, D’Orazio et al. developed chitosan-coated alginate microcapsules for *B. animalis* subsp. lactis PBS075, *L. rhamnosus* PBS070, and *L. plantarum* PBS067 (147). They found that in SGF and other adverse conditions, the encapsulated probiotics showed significantly higher resistance.

## Biomaterials for the Delivery of *Lactobacillus rhamnosus*

*L. rhamnosus* has been extensively studied since the 1980s. This species has a certain tolerance to gastric acid and bile. By adhering to intestinal cells, it can colonize the human body and has been shown to exert a variety of effects, such as reducing cholesterol levels, inhibiting α-glucosidase activity, and antioxidant and anti-inflammatory effects (152). LGG, a strain of *L. rhamnosus*, can reduce the expression of some inflammation markers and increase the levels of tumor necrosis factor-α, IL-10, and IL-12 in the macrophages to promote a type 1 immune response (153).

Its encapsulation and delivery improved immune regulation. Huq and coworkers developed alginate-cellulose nanocrystals (CNC)-lecithin microbeads for *Lactobacillus rhamnosus* ATCC 9595 (135) and found that CNC-lecithin microbeads had higher compression strength than alginate microbeads alone. Additionally, lecithin reduced the damage to bacterial membranes caused by bile salts in the intestine, thereby protecting probiotics. Another study prepared core-shell alginate–silica microcapsules for LGG (154). After the ionogels were formed using LGG and alginate, a silica coat was applied *via* a mild reaction process. In addition to enhancing the viability of the encapsulated LGG, the mesopores in the silica shell prevented bacterial leakage and allowed the diffusion of nutrient metabolites, thus ensuring bacterial growth within the microcapsules.

Because of considerably low pH levels in the gastrointestinal tract, the development of pH-responsive carriers for the protection and controlled release of bacteria in the stomach and intestine is necessary. Based on this view, an ethylenediaminetetraacetic–calcium–alginate (EDTA–Ca–Alg) system for *L. rhamnosus* ATCC 53103 was prepared using emulsification (137). In the acidic environment of the stomach, the structure of hydrogels remained intact and provided protection for the encapsulated bacteria. However, in neutral pH, as the EDTA completely chelates Ca^2+^, Ca^2+^ was released from the hydrogel structure, leading to its gradual disintegration, which was kept in a soluble state, thus allowing the release of bacteria.

## Biomaterials for the Delivery of *Escherichia coli*

*Escherichia coli* is a gram-negative facultative anaerobe and is one of the most characterized model organisms (155). *E. coli* is not only an extensive intestinal symbiont of vertebrates, but also a multifunctional pathogen. Therefore, using the excellent symbiotic characteristics of *E. coli* and technology, such as genetic engineering or plasmid transfection, *E. coli* can serve as a vector to carry target gene for the stable and efficient expression of specific metabolites, such as monoclonal antibodies (156) and enzymes (118), *in vivo*.

Chowdhuri et al. encapsulated *E. coli* using the inverse-emulsion technique to generate unilamellar vesicles (GUV) and verified the protective effect of liposomes on bacterial viability and activity (138). They demonstrated that *E. coli* encapsulated in liposomes could be protected from degradation by proteases in the stomach by prolonging their dissolution under acidic conditions. In addition, alginate–chitosan–alginate (ACA) microcapsules, which showed strong resistance against enzymatic digestion, were developed for the oral delivery of live bacterial cells for therapy (139). In this study, *E. coli* DH5 were encapsulated in ACA microcapsules and exhibited normal survival and growth during its passage through the stomach and intestine. This is due to the stability of ACA microcapsules in SGF.

## Biomaterials for the Delivery of *Lactobacillus plantarum*

*Lactobacillus plantarum* is a lactic acid bacteria with numerous strains, such as *L. plantarum* 80, *L. plantarum* NCIMB 1193, and *L. plantarum* Hu (157). *L. plantarum* can secrete SCFAs, especially butyric acid and acetic acid, and strengthen the intestinal barrier by enhancing epithelial defense (158). In addition, due to its ability to produce various effective bacteriocins (antimicrobial peptides), *L. plantarum* has been extensively used as a food preservative and antibiotic supplement (159).

Li and co-workers used cellulose microgels (CMs) to encapsulate *L. plantarum* (140). The CMs with their porous structure were shown to have an improved ability of carrying bacteria. Conjugation with alginate contributed to better resistance to acidic conditions and enhancement of the survival of bacteria. In 2017, Dafe et al. synthesized pectin-starch hydrogels to encapsulate *L. plantarum* ATCC:13643 cells using the extrusion method (126). By mixing different concentrations of pectin (2, 1.5, 1, and 0.5% wt) and starch (0, 0.5, 1, and 1.5% wt), hydrogels were divided into 4 groups, named Pectin, Pectin/starch1, Pectin/starch_2_, and Pectin/starch_3_, respectively. Compared with unencapsulated bacterial cells, those encapsulated in all ratios of pectin–starch hydrogels were found to be highly resistant to SGF solutions and showed higher survival rate. Chen et al. prepared alginate–poly-L-lysine–alginate (APA) microcapsules for L. plantarum 80 (LP80) (160). However, APA microcapsules only provided short protection in SGF. Upon exposure to an acidic environment (pH = 2) for 5 min, 80.0% of the embedded bacterial cells remained viable. However, after 15 min and 1 h, bacterial viability significantly decreased to 8.3% and 0.2%, respectively. These results represent the limitations of APA microcapsules for the oral delivery of live bacteria. To address these, Nualkaekul et al. designed alginate beads coated with chitosan for *L. plantarum*. Their approach enhanced bacterial resistance to both SGF and highly acidic pomegranate juice and increased bacterial survival (142). Furthermore, the multilayer chitosan coating of alginate beads improved their protective function, which increased with the number of coatings.

**Figure 6:**
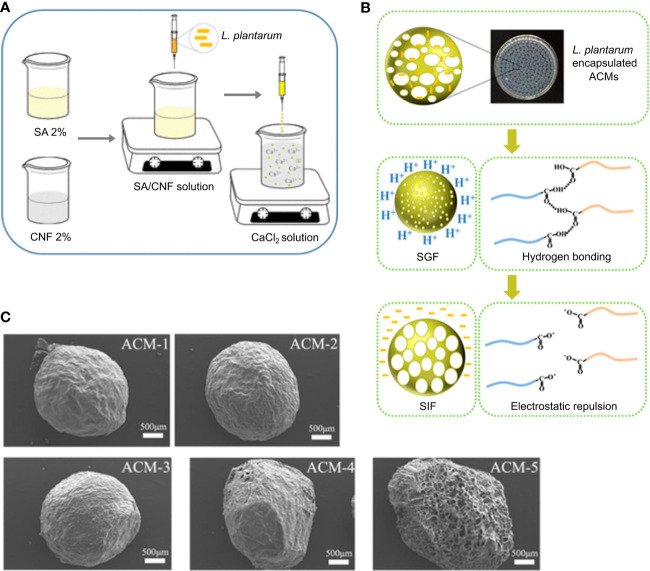
PH-responsive ACMs designed for delivery and release of *L. plantarum*. Overview of **(A)** the process of ACMs embeding *L. plantarum* and **(B)** ACMs designed for protecting *L. plantarum* against SGF and releasing in SIF. **(C)** SEM images of the freeze-dried ACMs. Adapted from ref. (161).

To prepare sodium alginate/cellulose nanofiber gel macrospheres (ACMs), Zhang et al. extruded a mixture of TEMPO-oxidized cellulose nanofibers (CNF) and sodium alginate (SA) within a CaCl2 solution (**Figure 6A**) (161). To further study the influence of the ratio of SA and CNF on the macrospheres, microspheres were prepared with the ratios of SA and CNF as 1:0, 3:1, 1:1, 1:3, and 0:1, and named them ACM-1, ACM-2, ACM-3, ACM-4, and ACM-5, respectively. After encapsulating the probiotics, *L. plantarum*, ACMs were placed in SGF to simulate the acidic environment in the stomach. After 2 h in SGF, these macrospheres were found to shrink and provide protection for *L. plantarum*, which might be because of the decrease of electrostatic repulsion causing by the protonation of carboxylic chains in CNF and SA (**Figure 6B**). When placed in SIF, *via* deprotonation of carboxylic chains and eliminating intermolecular hydrogen bonds, the ACMs swelled and finally ruptured to release *L. plantarum*, which targeted delivery of probiotics in the intestine (**Figure 6B**). Moreover, among the five different proportions of ACMs, with the proportion of increased CNF, the shrinkage of ACMs decreased in SGF and their rupture delayed in SIF, which demonstrated that CNF improved the stability of ACMs and SA was used for supplying pH-responsive function. **Figure 6C** shows the SEM images of ACM1 to ACM5 after freeze-drying.

In addition to these synthetic materials, some natural substances have also exhibited good loading capacity of bacteria. For instance, naturally occurring cavities and channels can be found in corn starch. After being expanded by enzymatic hydrolysis, natural corn starch can accommodate probiotics. Based on this property, Li and co-workers used fungal α-amylase (FA), pancreatin (P), and pancreatic α-amylase (PA) to partially hydrolyze corn starch and encapsulate *L. plantarum* 299v (141). Furthermore in using different enzymes, corn starch was treated with each enzyme for different times (30 and 120 min), with the materials after treatment being referred to as FA30, FA120, P30, P120, PA30, and PA120, respectively. These differently treated bacteria-containing corn starch samples showed a better acidic resistance, bile salt resistance, and survival rate, as well as exhibited an elevated delivering efficiency compared with free bacteria.

## Biomaterials for the Delivery of *Lactobacillus casei*

*Lactobacillus casei*, a gram-positive stain, is one of the most widely studied strains applied as fermentation starter in cultures (162) and probiotics (163). Due to its health-promoting properties, *L. casei* has been extensively researched. It was reported to effectively lower blood pressure and cholesterol, enhance human immunity, and inhibit or even prevent tumor growth (164, 165).

**Figure 7:**
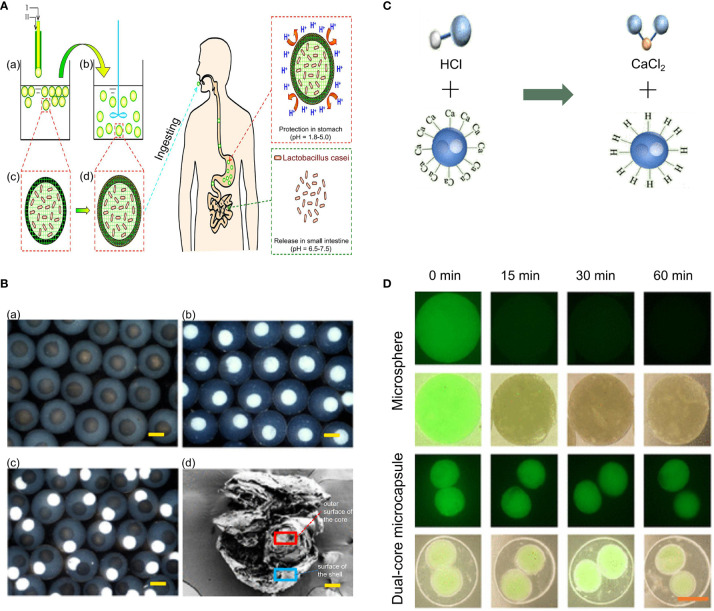
CAP beads and dual-core microcapsules prepared for encapsulating *Lactobacillus casei*, *Bacillus subtilis*, and *Lactobacillus* (NO. 21790). **(A)** Schematic illustration of the preparation process and the design concept of the proposed intestinal-targeted CAP carrier for the pH-responsive protection and release of *L. casei*. (a, c) CA beads prepared by a coextrusion method. “A” is Na-alginate solution containing *L. casei*, whereas “B” is pure Na-alginate solution. (b, d) CAP beads prepared by adsorption of protamine molecules. (e) Ingestion of CAP beads. (f) CAP beads offer improved protection to *Lactobacillus* in the stomach. (g) CAP beads rapidly release *L. casei* in the small intestine. **(B)** Characterization of microcapsules. optical microscope images of (a) the *Lactobacillus* microcapsules, (b) the Bacillus Subtilis microcapsules, and (c) dual-core microcapsules. (d) SEM images of dual-core microcapsules. Scale bars are 100 μm. **(C)** Alginate neutralizes HCL through metathesis reaction. **(D)** Detection of the activity of bacteria embeded in microspheres and dual-core microcapsules in SGF *via* fluorescent staining. The scale bar is 100 μm. Adapted from ref. (127, 166).

To efficiently deliver *L. casei*, Li et al. designed a novel intestinal targeting carrier for the anti-acid protection of *L. casei* and its controlled releasing in the gastrointestinal tract **(** **Figure 7A**) (127). First, calcium alginate (CA) beads were manufactured using a coextrusion mini-fluidic method of combining pure Ca-alginate solution and Na-alginate solution containing *L. casei*. Then, by employing an adsorption method, the prepared CA beads were adsorbed by protamine molecules to form calcium alginate/protamine (CAP) beads. In addition, sodium caseinate (SC) and lately developed SGF-resistant fat SC (FSC) capsules were also reported to significantly increase the survival of *L. casei* when passing through the upper gastrointestinal tract (129). Although *in vivo* experiments revealed that both SC and FSC capsules were eventually digested in the stomach 3 or 24 h after oral delivery, a high buffer capacity and good emulsification properties were still showed, making them a very suitable encapsulating materials with good research potential. Moreover, de Barro and co-workers produced dried live probiotic spheres (DLPS) of *L. casei* and mixed them with microcrystalline cellulose (MCC), calcium cross-linked alginate, and lactose followed by granulation (128). Subsequently, a Eudragit coating was added using an extrusion method to protect bacteria from acid and maintain the integrity of the entire structure in the acidic environment of the stomach. When reaching the intestine, increase in the pH led to the change of the Eudragit coating from hydrophobic to hydrophilic and the concomitant release of the bacterial cells.

## Biomaterials for the Delivery of Other Bacteria

In addition to the intestinal bacteria mentioned above, there are also many other bacteria in the intestine that are known to have the function of promoting the health of the human body. To make the oral delivery of these intestinal bacteria more efficient, many suitable biomaterial schemes have been designed for their encapsulation.

Based on the yin–yang concept, microcapsules with independent internal compartments, which can encapsulate and deliver a variety of substances, such as drugs and microbes, to promote their functions without interference, have been designed (167, 168). Zhao and coworkers designed a dual-core microcapsule to encapsulate and deliver *B. subtilis* and *Lactobacillus* (NO. 21790) in separated microcompartments (166). First, each intestinal bacterium was mixed in a solution containing CNC, carboxymethyl cellulose (CMC), and sodium alginate. Then, it was electrosprayed to prepare the microspheres (containing only one kind of intestinal bacteria). In this technique, CMC had the function of neutralizing HCl to protect bacteria in an acidic environment, while CNC could restrict these bacteria to a certain range *via* depletion flocculation. Aluminum chloride (AlCl_3_) was the chosen crosslinking agent as it caused the microspheres that encapsulated the intestinal bacteria to form quickly. Subsequently, the outer phase fluid could be wrapped around the inner core by hydrodynamic focusing, and the alginate could be quickly solidified after the solution containing the dual cores was placed in the presence of electric field in a gel bath with 2% CaCl2, thereby forming the dual-core microcapsules. **Figure 7B** shows the optical microscope and SEM images of dual-core microcapsules. To further study the protective effect of dual-core microcapsules on bacteria in an acidic environment, dual-core microcapsules and microspheres without alginate shells were placed in simulated gastric juice (SGF) for 60 min to observe the bacterial activity. Theoretically, the dual-core microcapsules could provide protection to the encapsulated bacteria in an acidic environment because of the sodium alginate shell, which could neutralize HCL through a metathesis reaction (**Figure 7C**). As shown in **Figure 7D**, compared with dual-core microcapsules, intestinal bacteria encapsulated in microspheres without alginate shells lost most of their activity in the first 15 min. However even after 60 min, more than 70% of the probiotics encapsulated in dual-core microcapsules retained their activity in SGF.

**Figure 8:**
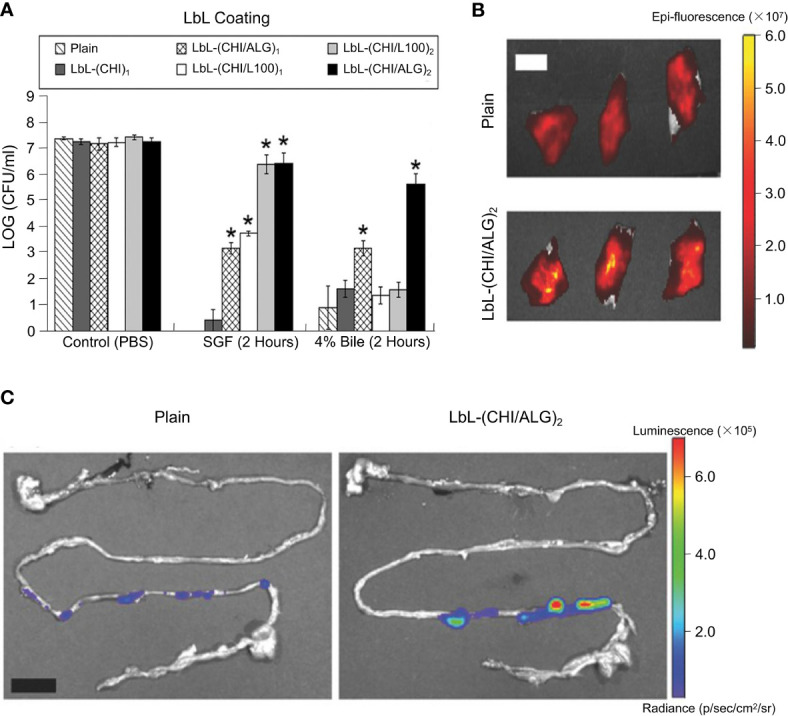
Layer-by-layer encapsulation of probiotics employed to enhance their survival rate against acidic and bile conditions, and their physical retention in the intestine. **(A)** Schematic LbL encapsulation of chitosan and alginate on probiotic. **(B)** LbL formulated (CHI/ALG)_2_ (black bars) BC were protected against both acidic and bile salt conditions at 37°C for up to 2 h. LbL coatings of chitosan (dark gray bars), (CHI/L100)_1_ (white bars), (CHI/L100)_2_ (light gray bars), and (CHI/ALG)_1_ (cross-hatched bars) were less effective at protecting BC against both acidic and bile conditions. Error bars represent standard deviation (n = 3). *denotes statistical difference (P < 0.05) using Student’s *t*-test between plain and LbL groups. **denotes statistical difference (P < 0.05) using individual Student’s *t*-test between the designated and any other group. **(C)** IVIS images of porcine intestine with plain- and (CHI/ALG)_2_-probiotics. Adapted from ref. (143).

Anselmo et al. combined chitosan (CHI) with alginate (ALG) as one CHI/ALG bilayer and then encapsulated intestinal bacteria using LbL method (**Figure 8A**) (143). In their study, *Bacillus coagulans* was used as a model strain. Several different LbL formulations and numbers of layer were designed to research their protecting function. An enteric polymer, L 100, was chosen to encapsulate probiotics in combination with chitosan. Compared with *B. coagulans* encapsulated using chitosan only, L100 combined with chitosan (denoted as CHI/L100) was able to protect the probiotic in SGF but failed against bile (**Figure 8B**). *B. coagulans* encapsulated by 2 CHI/ALG bilayers, expressed as (CHI/ALG)_2_, was reported to be well protected against both SGF and bile (**Figure 8B**). **Figure 8C** shows that bacteria encapsulated by (CHI/ALG)_2_ presented higher adhesion to the intestine in slices of freshly isolated small intestine. Additionally, the (CHI/ALG)_2_ coating was shown to improve the survival of probiotics in the intestine. Ouyang and co-workers improved the APA microcapsules and designed multilayer alginate/poly-L-lysine/pectin/poly-L-lysine/alginate (APPPA) microcapsules to encapsulate *L. reuteri* cells (144). Stability of APPPA microcapsules were tested as well as the activity of encapsulated bacterial cells at 37.2°C under various pH conditions. When microcapsules were placed into SGF and SIF, respectively, for a total of 24 h (12 h in SGF and 12 h in SIF) at 250 rpm mechanical shaking at 37.2°C, no obvious damage to bacterial cells was reported. After 24 h in GSF and SIF, respectively, more than 90% of APPPA microcapsules remained intact, exhibiting a good resistance to mechanical shocks. Compared with APA microcapsules, APPPA microcapsules showed an excellent stability. The reason behind this was the use of poly (amino acid), which is a material that has been widely used for encapsulating bacteria. It was therefore assumed that using more poly-L-lysine to encapsulate bacteria would have a better protection and delivery effect. Besides, when designing biomaterial for encapsulating bacteria, the number of layers is also considered to be an important factor affecting delivery efficiency. Accordingly, the effect of this protection was proportional to the number of layers in a certain range, rather than with the increasing in the number of layers (146).

## Biomaterials for Delivering Bacterial DNA

Many intestinal bacteria can regulate the immune function and promote host health mainly *via* their DNA (169). Therefore, the encapsulation and delivery of bacterial DNA has great potential. As a positively charged natural polymer, chitosan can entrap nucleic acids (NA) (both RNA and DNA) and protect them from degradation by nuclease (170). However, chitosan is rarely used as NA carrier because of its poor water solubility and low transfection efficiency. To overcome these limitations, various modifications and formulations have been proposed (171, 172). For instance, Zhang et al. developed the PEGylation of chitosan nanoparticles for the delivery of DNA. These chitosan–DNA–PEG complexes increased the dose of DNA delivered and have been indicated to exhibit a liver tumor targeting (173). In another study, chitosan salts, such as chitosan glutamate, chitosan aspartate, chitosan acetate, chitosan lactate, and chitosan hydrochloride, were used to form chitosan–DNA complexes (174). Compared with standard chitosan, these complexes showed better transfection efficiency and lower cytotoxicity.

## Conclusion and Prospects

The regulation of intestinal bacteria to enrich target probiotics might be an effective strategy to improve the immunotherapeutic response. However, the best way to achieve this goal remains to be determined. When intestinal bacteria are transplanted as a whole (such as in FMT), thousands of bacterial species are simultaneously introduced. As such, this approach is not conducive to the in-depth study of the functions of each bacterial species. In addition, although the combination of intestinal bacteria in FMT has been shown to be of vital importance in this treatment, FMT can affect the abundance of main bacteria, while other bacteria do not exhibit any effect or even reduce the function of the main probiotic. Even if we could isolate the main bacteria, the immunomodulatory effects of the same strain would not appear to be completely consistent in different diseases and different studies. As we mentioned earlier, *Lactobacilli* populations showed diametrically opposite regulatory functions for Tregs in different studies. Therefore, we considered that the regulation of immunotherapy by intestinal bacteria could be affected by other factors.

Early in 2006, Hara et al. considered intestinal bacteria as a whole and proposed them as a potential organ of the human body (175). When artificially enriching or eliminating the number of intestinal bacterial species, this is generally accompanied by the reestablishment of a new multibacterial environment. This significant change in the composition of intestinal bacteria might explain the significant differences observed in the role of certain bacteria and might explain the positive effects of regulating intestinal bacteria on the efficacy of disease immunotherapy. In addition, intestinal bacteria are also known to be interdependent with the immune cells that they regulate. For example, *Bifidobacterium* was reported to improve the effect of PD-L1 inhibitor in antitumor treatment by regulating cytotoxic T-cells (15). However, *Bifidobacterium* could not exhibit this immunomodulatory effect in cytotoxic T-cell-depleted mice, suggesting that its effect was depended on the activity of cytotoxic T-cells. More specifically, it was assumed that intestinal bacteria serve more as aggregators or amplifiers of immune cells, which can in turn enhance the efficacy of immunotherapy. However, this regulatory function was demonstrated to rely on the adequacy of immune cells and the integrity of the immune system. Besides their interaction with immune cells, intestinal bacteria have also been shown to avoid or alleviate pathological conditions in various diseases, such as nonspecific inflammation. For example, Foxp3^+^ Tregs were found to be widely involved in the immunotherapy of various diseases. This was attributed to the role of Foxp3^+^ Tregs in affecting immune responses and suppressing the progress of inflammation. Based on this feature, several probiotics and the PSA of *B. fragilis* have been used to inhibit the progression of inflammation through the upregulation of Foxp3^+^ Tregs and to promote the immunotherapy of IBD (70) or the HIV-1 infection (67). Therefore, focusing on these common beneficial effects of intestinal bacteria and trying to combine them in treatment applications against various diseases or clinical problems might greatly expand the application prospects of intestinal bacteria.

The regulation of immune cells by intestinal bacteria is known to be accompanied by other substances in the microenvironment, cytokines, which can recruit and activate different kinds of immune cells. Many of the studies mentioned above have shown that the levels of interleukins change following the administration of intestinal bacteria. Independent of being upregulated or downregulated, the correlation between intestinal bacteria and interleukins has revealed the mechanism of the immune regulation by intestinal bacterial to a certain extent, and has also shown the feasibility of the combined use of these 2 factors for the immunotherapy of various diseases. Apart from interleukins, the levels of chemokines (75) and interferons (15) in the body have also been shown to be affected by intestinal bacteria. In particular, upregulation of IFN-γ by intestinal bacteria (such as *Bifidobacterium*) has also revealed to a certain extent the reason behind the ability of intestinal bacteria to promote the effect of ICB therapy in tumor immunotherapy. Recent studies have found that IFN-γ could upregulate the production of PD-L1 and establish the IFN-γ/PD-L1 axis based on the relationship between them (176–178). Furthermore, IFN-γ was found to exhibit the same effect on the expression of CTLA-4 (179, 180). Respectively, administration of intestinal bacteria to increase the expression of PD-L1 and CTLA-4, would undoubtedly greatly improve the effect of anti-CTLA-4 and anti-PD-1/PD-L1 therapies. Therefore, selecting the appropriate cytokines to synergize with intestinal bacteria could greatly enhance the effect of immunotherapy and the regulatory function of intestinal bacteria. Not only limited to cytokines, this synergy with intestinal bacteria established by immune cells could lead to more research ideas.

Regarding the intestinal bacteria used to regulate immunotherapy, there are also some problems that need in-depth study. One concern is the duration of their regulation of the immune system. As we know, intestinal bacteria are specific to people living in different areas or enjoying different diet habits, similar to our physical characteristics, and this specificity is known to determine the sensitivity of everybody to diseases and immunotherapy. As mentioned earlier, because of the differences in the composition of intestinal bacteria, patients who suffered from melanoma and were treated with PD-1 inhibitor were divided into responders (R) and non-responders (NR). This phenomenon implied that as long as the composition of intestinal bacteria remains stable in the body, their regulatory function might be long-term. Based on this view, intestinal bacteria could not only be used to regulate immunotherapy but might also be a major factor to prevent the diseases. Unlike conventional vaccines or vaccines based on genetically engineered bacteria, this kind of disease prevention *via* the direct use of intestinal bacteria might exhibit better compliance and longer-term preventive effects. Moreover, with deeper studies on their ability for immune regulation, it might be entirely possible to select the most suitable multi-bacterial environment and reconstruct it in unaffected or affected hosts by introducing various probiotics in order to achieve the prevention of many diseases and the maintenance of health status.

However, we would also need to consider how this multi-bacterial commensalism can be established and made stable in the body. First of all, in order to improve compliance and avoid infections caused by bacteria entering the blood, oral delivery of intestinal bacteria should be routinely selected. Second, in order to ensure the vitality of extraneous intestinal bacteria, these should be delivered in the form of microcapsules, that is, bacteria should be encapsulated into microspheres. Therefore, related encapsulation materials and technologies have to be developed to ensure the protection of bacteria from the acidic environment in the stomach and their complete release in the intestine. Furthermore, following the introduction of encapsulated bacteria in the body and the establishment of a new multi-bacterial environment, it would be necessary to maintain this multi-bacterial environment in the long term. As we all know, diet is an important factor in the composition of the intestinal bacteria. Therefore, providing an appropriate diet is an important method to this end. However, its precondition is the knowledge of the relationship between diet and intestinal bacteria. Hence, immunotherapy for many diseases, including tumors, is expected to open a new chapter through the dietary regulation of intestinal bacteria to achieve the prevention of diseases, promotion of immunotherapy during diseases, and maintenance of the curative effect after the resolution of diseases.

In this review, we have outlined the potential use of intestinal bacteria for the regulation of immunotherapy of diseases and the useful biomaterials employed for encapsulating these bacteria. Future studies should focus on developing efficient encapsulation and delivery methods and accurate targeting ability and establishing stable multi-bacterial environments to expand the clinical applicability of intestinal bacteria in disease treatment. In addition, dietary factors and genetic engineering can provide more methods and possibilities for their clinical application. We believe that with the continuous in-depth studies of intestinal bacteria and exploration of their synergistic strategies, better, safer, and more effective intestinal bacterial therapies will be used in clinical practice with more people benefiting from them.

## Author Contributions

All authors contributed to the article and approved the submitted version.

## Conflict of Interest

The authors declare that the research was conducted in the absence of any commercial or financial relationships that could be construed as a potential conflict of interest.

The reviewer XW declared a shared affiliation with several of the authors YL, ZL, YW, LL, XJ, XF, to the handling editor at time of review.
